# Influence of Lead-Free Perovskite Panels on Indoor Growth of *Solanum lycopersicum* L. and *Artemisia annua* L. Plants

**DOI:** 10.3390/plants14203195

**Published:** 2025-10-17

**Authors:** Sofia Caretto, Angelo De Paolis, Annalisa Paradiso, Francesco Milano, Bruno Olivieri, Carlo Ottaviani, Paola Prete, Paola De Padova

**Affiliations:** 1 Istituto di Scienze delle Produzioni Alimentari—CNR, Via Monteroni, 73100 Lecce, Italy; angelo.depaolis@cnr.it (A.D.P.); annalisa.paradiso@cnr.it (A.P.); francesco.milano@cnr.it (F.M.); 2 Istituto di Scienze dell’Atmosfera e del Clima—CNR, Via Fosso del Cavaliere, 100, 00133 Roma, Italy; b.olivieri@isac.cnr.it; 3 Istituto di Struttura della Materia—CNR, Via Fosso del Cavaliere, 100, 00133 Roma, Italy; carlo.ottaviani@ism.cnr.it; 4 Dipartimento di Ingegneria dell’Innovazione, Università del Salento, Via Monteroni, 73100 Lecce, Italy; paola.prete@unisalento.it; 5 Istituto Nazionale di Fisica Nucleare—Laboratori Nazionali di Frascati, via Enrico Fermi, 54, 00040 Frascati, Italy

**Keywords:** perovskite panel, optical properties, plant growth, *Artemisia annua* L., *Solanum lycopersicum* L., photosynthetic pigments

## Abstract

This work focuses on research into innovative lead-free perovskite materials to be employed as a sensitive layer for a new generation of solar cells, exploiting their potential applications in covering greenhouses to move toward an eco-friendly environment. Two types of lead-free perovskites—yellow and orange double-cation Cs_2_AgBiBr_6_, synthesized with an innovative method without chemical thinners—have been used, for the first time, as a cover for greenhouses in indoor experiments by analyzing the incident electromagnetic radiation. Two plant species, *Solanum lycopersicum* L. and *Artemisia annua* L., were cultivated indoors under controlled light, temperature, and humidity, covering the greenhouses with yellow (PY+) and orange (PO+) panels for comparison with control plants (P−) roofed by a glass panel. The growth and development parameters of all plants were investigated, referring to the aerial and root parts. Significant differences were found in terms of the plant growth parameters and photosynthetic pigments of both PY+ and PO+ compared to P− and also between them, with the yellow panel being less invasive. These results, dealing with two different plant species, confirm the feasibility of using perovskite-based panels for indoor cultivation and pave the way for outdoor application in greenhouses under sunlight.

## 1. Introduction

Increasing demand for sustainable solutions for energy production can be found in many production sectors. Food production by agriculture activities is certainly one case, due to the high amounts of energy needed to manage crop cultivation. Photovoltaic systems, producing electricity from solar light, are optimal sources of renewable energy, reducing environmental impacts such as carbon dioxide emissions and thus mitigating climate change impacts [1,2]. However, photovoltaic panels have often been used at the expense of cultivated land, reducing crop production and food security. A recent study reported that between 13% and 16% of current ground-mounted photovoltaic plants are located on agricultural land that is no longer cultivated [3]. Investigating sustainable solutions that allow the production of renewable energy and, at the same time, safe cultivation of crops is a priority. In this regard, the double use of land for both crop production and solar energy generation can enhance net income and resource efficiency, making it an attractive option for farmers [4].

The possibility of exploiting sunlight to generate electricity, while at the same time allowing the healthy growth of plants (also protecting them from the harmful excess of light), could be achieved by covering greenhouses with semitransparent solar panels. An essential condition is that light radiation passing through the panels enables photosynthesis to be carried out by plants. Perovskite-based halide solar cells appear to be an ideal candidate to achieve this goal. Furthermore, lead-free perovskite can be an especially environmentally friendly solution [5,6].

Perovskite solar cells (PSCs) are presently a promising emerging photovoltaic technology due to both their highly efficient sunlight energy conversion and their low cost of synthesis. Since the first discovery of the organometal lead halide perovskites CH_3_NH_3_PbBr_3_ and CH_3_NH_3_PbI_3_ as photo-absorbers for photovoltaic (PV) cells by Kojima et al. in 2009 [7], the initial power conversion efficiency (PCE) of 3.13% and 3.81% has had an unprecedented progression of up to 25% in only a few years, to a high value of more than 32% when organized in a tandem multijunction [8,9], becoming competitive with PV silicon technology. As a consequence, this has enormously promoted increasing interest in both academic and social frameworks. Perovskites are ABX3-type structures, where A can be rubidium (Rb), cesium (Cs), methylammonium (MA), and/or formamidinium; B tin (Sn) and/or lead (Pb); X chlorine (Cl), bromine (Br), and/or iodine (I). The band gap of these perovskites can be tuned from 1.15 to 3.06 eV by interchanging cations, metals, and/or halides/mixed halides [10], while mixtures of Sn and Pb metals move the band gaps from 1.59 eV (MAPbI3) to 1.29 eV (MASnI3). On the other hand, increasing the amount of Sn can further result in band gaps narrowing to 1.2 eV, close to the silicon one.

Toxicity of Pb compounds and losses of structural stability of organic–inorganic perovskites are the major restrictions to their exploitation in anthropological activity and/or commercial applications [11,12]. This is mainly due to the mandatory request for the preservation of human and environmental health, in the framework of the worldwide green deal. One strategy is to move toward developing Pb-free perovskites, to increase their stability and durability over time, while providing high light conversion power. In fact, completely inorganic Pb-free halide double perovskites, having a typical chemical formula A_2_BB’X_6_ (X = Cl, Br) based on Bi and Ag, have recently been synthesized as highly promising alternatives to hybrid halide Pb perovskites, exhibiting suitable band gaps in the entire visible spectrum [6,13,14,15,16].

The yield response to shading effects of solar panels can be variable in different plant species, either cultivated for biomass or food production; some crops take advantage of the reduced solar radiation, while others undergo yield losses even at low shading levels. Also, physiological and biochemical parameters related to photosynthesis in response to shading effects can be variable in different crops, as a consequence of specific adaptation to reduced light conditions [17].

Tomato (*Solanum lycopersicum* L.) and Artemisia (*Artemisia annua* L.) are two plant species, both largely used by humans, though for different purposes. Tomato is a crop plant belonging to the Solanaceae family, widely cultivated throughout the world, and well known for its nutritional value [18]. Tomatoes represent approximately 22% of the total world vegetable supply [19]. Tomato plants are known to contain many bioactive metabolites, particularly in fruit tissues. Among them, carotenoid and phenolic compounds are the most studied [20,21]. Artemisia is an officinal plant, belonging to the Asteraceae family, that has been used by traditional Chinese medicine for many years to treat various diseases, including fever caused by malaria [22]. The later discovery of the sesquiterpenoid artemisinin, which is highly effective as an antimalarial drug, increased the interest in Artemisia, which is nowadays also known for many other bioactive compounds, including isoprenoids, flavonoids, coumarins, and phenolics, responsible for anti-inflammatory and anti-cancer activities [23,24,25].

In view of a potential contribution to agricultural sustainability, energy utilization efficiency, and environmental protection, this work aimed to evaluate the suitability of two different types of semitransparent, yellow and orange, perovskite panels for covering greenhouses, used, for the first time, for growth and development of two plant species, such as tomato and Artemisia cultivated indoors under controlled conditions, including the use of a controlled and reproducible light source. Semitransparent panels were realized using yellow and orange Cs_2_AgBiBr_6_ perovskites synthesized with a completely “green” innovative and unique procedure, without the use of chemical solvents [26]. The optical properties, through transmittance, reflectance, and absorbance measurements, in the range of wavelengths from ultraviolet (UV) to infrared (IR) of yellow and orange Cs_2_AgBiBr_6_ perovskites have been reported, highlighting the insights revealed by plant responses to the changed environmental conditions due to the covering of perovskite panels.

## 2. Results and Discussion

Unlike conventionally used opaque solar panels, in this work, two types of semitransparent panels characterized by different colors, yellow (PY+) and orange (PO+), were used. Although the panels used in the experimental phase were electrically non-functional, they were made up of all the essential functional layers, faithfully mimicking the transmittance of real panels. This structure made it possible to investigate the impact on plant growth and physiology in depth.

### 2.1. Optical Properties of Yellow and Orange Cs_2_AgBiBr_6_ Perovskite Panels, and In Situ Fluorescent Lamp Spectrum

**Figure 1:**
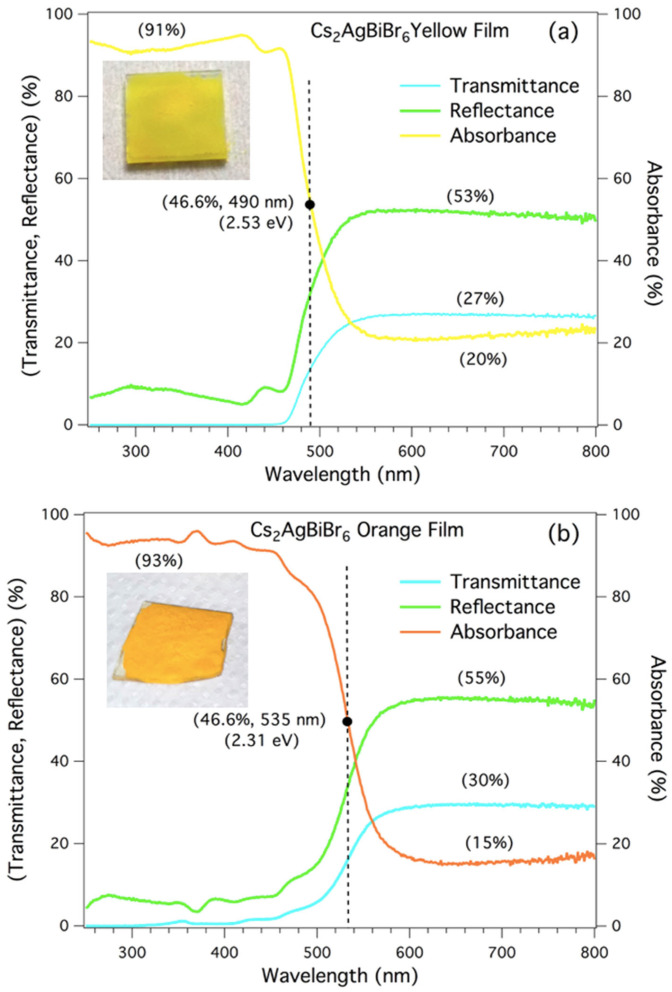
Transmittance, reflectance, and absorbance spectra, in (%), of the yellow (**a**) and orange (**b**) perovskite Cs2BiAgBr6 films, where the values (%) of the plateau at low frequencies of light (infrared-visible) are indicated: 20%, 27% and 53% for yellow, and 15%, 30% and 55% for orange, respectively. Optical images are reported, as an inset, for both yellow (**a**) and orange (**b**) perovskites.

The transmittance, reflectance, and absorbance spectra, in %, in the range of 250 nm to 800 nm, are reported in Figure 1, from yellow (a) and orange (b) perovskite Cs_2_BiAgBr_6_ films. The absorbance curve is obtained from the transmittance and reflectance spectra (100-transmittance-reflectance).

The optical images for both yellow (a) and orange (b) perovskites are reported as an inset. For the spectra shown, the dark point is placed on the absorbance curve to indicate its inflection point. A straight dashed line is then drawn vertically from this point. This line crosses the inflection points of the transmittance and reflectance curves, which all coincide at the same wavelength but occur at different y-axis values. These shared wavelengths are located at 490 nm (2.53 eV) for yellow perovskite (a) and 535 nm (2.31 eV) for orange perovskite (b). These values signal the wavelength at which the slope of the curves changes due to modified optical properties. These perovskites are compounds, exhibiting electronic properties with an indirect gap of about 1.95 eV, and a larger direct gap of about 2.3 eV, presenting an energy range that can vary from 1.6 eV up to about 2 eV, depending on the presence of defect states in the gap or doping atoms [6,13,14,15,16,26,27,28,29,30,31,32].

Percentage values of the plateau, at low frequencies of light (infrared-visible), are indicated: 20%, 27% and 53% for yellow, and 15%, 30% and 55% for orange, respectively, resulting in not very different values. What is different is the inflection point of the two absorption curves, which differs by 45 nm, making the yellow perovskite more transmitting around the blue frequencies of visible, absorbing just 46.6% at 490 nm, whereas the orange absorbs at the same wavelength, about 80%.

**Figure 2:**
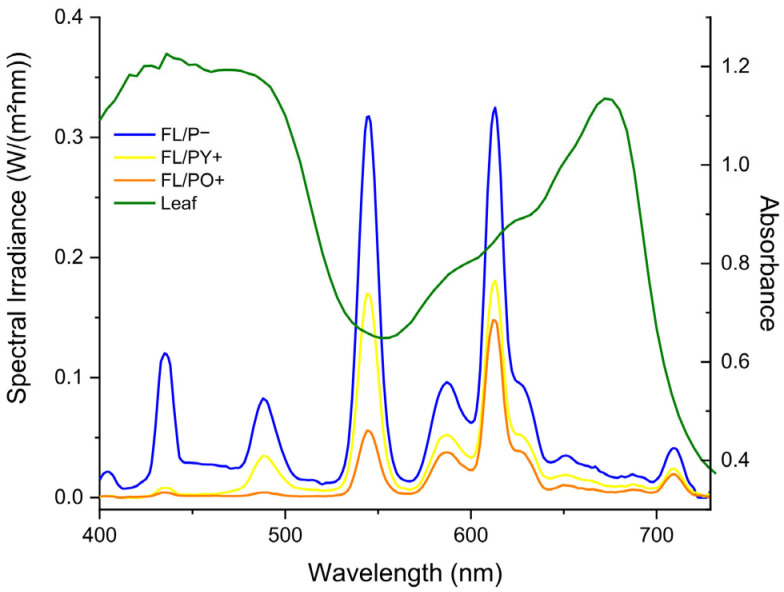
Irradiance spectra resulting from the fluorescent lamp, measured at 25 cm and filtered through a transparent glass panel (blue trace, P−), a yellow perovskite panel (yellow trace, PY+), and an orange perovskite panel (orange trace, PO+). The green trace shows the reflectance spectrum of a healthy green leaf.

Physical parameters, in terms of light spectrum of the light source and the interference due to the presence of the perovskite panels, were recorded with the spectral light meter MSC15 (Gigahertz Optik GmbH, Türkenfeld, Germany) and are reported in Figure 2. The bare fluorescent emission (black trace) features five main peaks at 435 nm, 488 nm, 545 nm, 587 nm, and 613 nm and a total irradiance of 5.00 W/m^2^ (PAR = 22.4 µmol/(m^2^s)). The presence of glass (blue trace), used as a cover for the mini greenhouses, had a minor impact on the light, with a total irradiance of 4.15 W/m^2^ (PAR = 18.7 µmol/(m^2^s)) and unchanged spectral profile. Yellow perovskite (yellow trace) completely absorbed the 435 nm emission peak and roughly halved the others, resulting in a total irradiance of 2.06 W/m^2^ (PAR = 9.60 µmol/(m^2^s)). Orange perovskite (orange trace) had a larger impact on the shorter wavelength peaks, completely absorbing the peaks at 435 nm and 488 nm, and reducing the peak at 545 nm by more than 80%. On the contrary, the peaks at 587 nm and 613 nm were slightly reduced as compared to the yellow perovskite. Total irradiance for orange perovskite was 1.14 W/m^2^ (PAR = 5.37 µmol/(m^2^s)).

It should be noted that the behavior of the spectra reported in Figure 1, for both perovskites, is perfectly in line with that reported in Figure 2, where the quantification in terms of radiant power through the yellow and orange panels is reported.

The spectral response of a healthy green leaf (green trace in Figure 2) is characterized by strong absorption within the visible part of the electromagnetic spectrum (400–700 nm), due to leaf pigments, mainly chlorophyll, absorbing at 450 nm (blue) and 650 nm (red). The valley at 510–580 nm corresponds to the visible green light, responsible for the characteristic leaf color. After a transition region from low to high reflectance termed “red-edge” (680–780 nm), the near infrared (NIR) region (700–1300 nm) is characterized by strong leaf reflectance due to back scattering caused by the plant cell structure (spongy mesophyll tissue, palisade tissue, protoplasm and chloroplasts within the leaves) [33,34].

Comparing the spectral features of green leaves and glass/perovskite filtered light, it can be inferred that both yellow and orange perovskite have an impact on the overall light intensity reaching the plants; however, the expected effect should not be as large as obtained by the total irradiance since the most affected peak falls at 545 nm, near the flex of absorbance curve of orange perovskite (Figure 1b), and 45 nm away from the yellow one (Figure 1a) at the reflectance peak, i.e., its energy is not being much used by the plants for photosynthesis. On the other hand, the second larger peak at 613 nm, located in a region with higher absorption by chlorophylls, is affected by the perovskite layers in a similar way.

### 2.2. Tomato Plants

Tomato plants (*Solanum lycopersicum* L., cv. Micro-Tom) were cultivated in the mini-greenhouses covered by semitransparent yellow (PY+) and orange (PO+) perovskite panels, and a transparent glass (P−), as described in Section 3.

**Figure 3:**
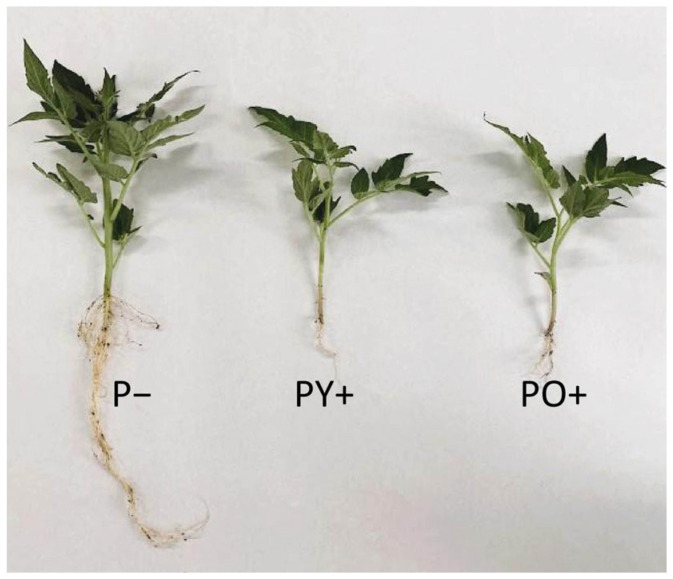
Tomato plants grown for 21 days under a transparent glass panel as control (P−), a yellow perovskite panel (PY+), and an orange perovskite panel (PO+).

Figure 3 shows the comparison between tomato plants which are representative of the three growth conditions after three weeks. The results of the plant growth parameters are reported in Table 1.

In general, tomato plants grown under the two perovskite panels showed significantly reduced growth compared to control plants; PY+ and PO+ plants showed significant differences between them only in the aerial part, with plant heights 23% and 40% lower than P− in PY+ and PO+, respectively. Plant growth measured as fresh weight of aerial parts was significantly reduced in both PY+ (−44%) and PO+ plants (−55%) compared to control plants. A similar reduction was also observed when considering DW.

The roots of plants grown under PY+ and PO+ panels were both strongly reduced compared to control plants, showing about 60% reduction in length and about 90% reduction in fresh and dry weight. No significant differences were evident between the PY+ and PO+ root growth parameters (Table 1). In plants grown under perovskite panels, root development appeared more inhibited than that of the aerial part, and, consequently, the aerial part/root ratio strongly shifted in favor of the photosynthesizing aerial part.

When the aerial/root ratio was calculated for the fresh weight of the tomato plants, it was 6.78 ± 0.55, 30 ± 3 and 40.2 ± 2.9 for P−, PY+ and PO+ plants, respectively. The ratio between the aerial part and the roots followed the same trend, also referring to the dry weight.

This higher level of aerial/root ratio of P+ plants could be attributed to the plant response to light depletion produced by the panel application. Plants could primarily distribute metabolic energy to leaves and stems, at the expense of roots [35,36,37]. On the other hand, it is known that light changes can significantly influence not only the aboveground plant canopy, but also elongation and morphology of roots [38,39,40].

Growth parameters in tomato plants under a transparent glass panel as control (P−), the yellow perovskite panel (PY+), and the orange perovskite panel (PO+) after 21 days are reported in Table 1 for the aerial/root part. Measured parameters for tomato plants are plant height, fresh and dry weight for the aerial part; length, fresh and dry weight for plant roots. Compared to control plants (P−), both PY+ and PO+ plants also showed reduced height and changed leaf parameters, as shown in Table 2, which reports the values of leaf number per plant, leaf area, and length of the fully developed leaf.

Leaf number, leaf area, and length of the fully developed leaf were significantly reduced in presence of both the perovskite panels. PY+ and PO+ plants differed significantly from each other only in leaf number.

**Figure 4:**
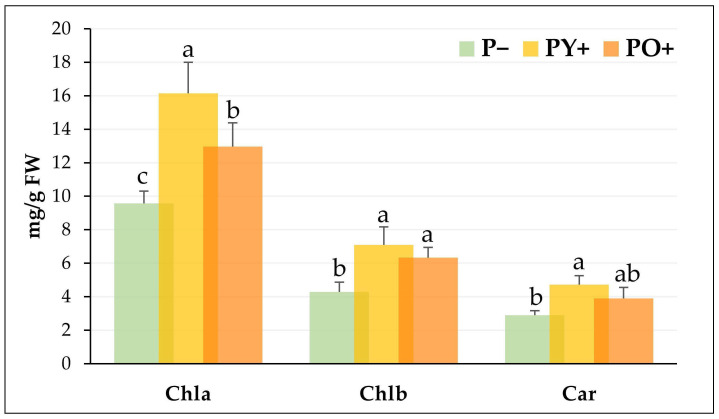
Chlorophyll (a and b) and carotenoid content of tomato plants grown under a transparent glass panel as control (P−), yellow perovskite panel (PY+) and orange perovskite panel (PO+). The bars show mean and standard deviations of five biological replicates with the use of the youngest fully developed leaves for each plant. Bars with different letters are significantly different at *p* ≤ 0.05 (Tukey test, *p* ≤ 0.05).

Chlorophyll a (Chla), chlorophyll b (Chlb), and carotenoids (Car) are paramount plant pigments, playing a crucial role in light harvesting and being structural components of the photosynthetic process. Since leaf photosynthetic pigments can be affected by the intensity of light and its spectral region, the analysis of their contents in tomato plants grown under P−, PY+, and PO+ was performed and is reported in Figure 4. The content of Chla and Chlb was significantly higher in plants grown under PY+ and PO+ panels than in control plants. Car content significantly increased only in plants grown under PY+. However, higher levels of Car, though not significant, were also detected in plants grown under PO+ panels. In the three growth conditions, the Chla/Chlb ratio was found to be stable within the experiment, ranging from 2.048 to 2.27; similarly, the (Chla + Chlb)/Car ratio remained stable from 4.78 to 4.95.

The observed increase in pigment content could be a compensatory response, due to the decrease in light intensity, to improve the utilization of light energy, as already reported [41,42]. On the other hand, the particularly noticeable drop in intensity in the green wavelengths could not interfere with photosynthetic activity, because the most useful wavelengths for photosynthesis are in the blue and red regions of the light spectrum.

### 2.3. Artemisia Plants

Artemisia plants were cultivated in the mini-greenhouses covered by semitransparent yellow (PY+) and orange (PO+) perovskite panels, and transparent glass (P−), as described in Section 3. Artemisia plants were collected three weeks after the beginning of the experiment, and analyzed for growth parameters and photosynthetic pigment contents.

**Figure 5:**
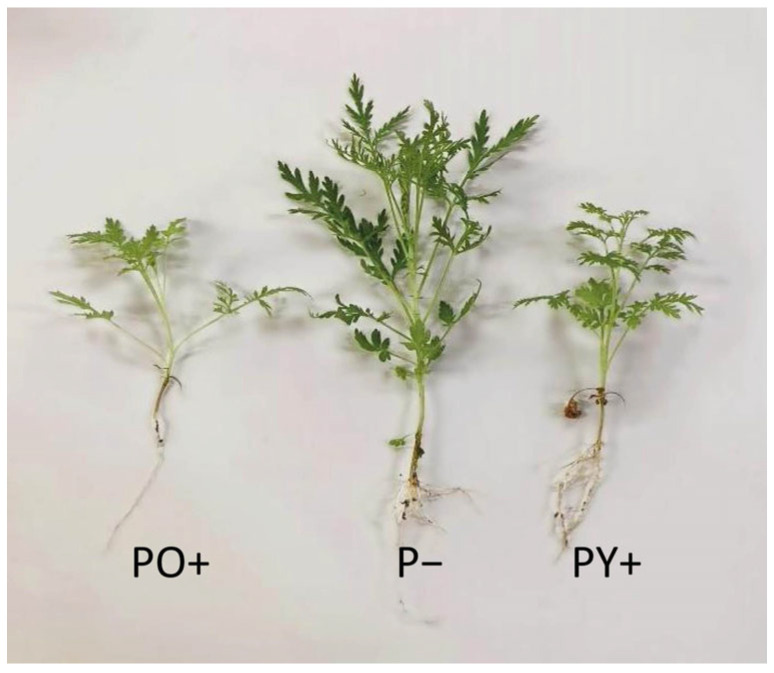
Artemisia plants grown for 21 days under a transparent glass panel as control (P−), a yellow perovskite panel (PY+) and an orange perovskite panel (PO+).

A comparison between the representative Artemisia plants grown under the three growth conditions showed a reduced development under PY+ and PO+ (Figure 5).

Table 3 reports growth parameters in Artemisia plants under a transparent glass panel as control (P−), a yellow perovskite panel (PY+) and an orange perovskite panel (PO+) after 21 days. Measured parameters for Artemisia plants are plant height, fresh and dry weight for the aerial part; length, fresh and dry weight for plant roots.

As shown in Table 3, plant growth, both at the aerial and root levels, was significantly reduced by the presence of both perovskite panels. Only the height of the plants significantly varied between the plants grown under PY+ and PO+ panels, showing a reduction of approximately 44% and 55%, respectively, compared to P− plants.

Table 4 reports leaf parameters per plant, including leaf number, leaf area and length of the fully developed leaf, in Artemisia plants grown under a transparent glass panel as control (P−), the yellow perovskite panel (PY+) and the orange perovskite panel (PO+) after 21 days. The decrease observed in fresh/dry weight of the aerial part of Artemisia plants grown under perovskite panels can be ascribed to the reduction in the values of leaf parameters (see Table 4). Plants grown under the perovskite panels had a lower leaf number (−34% and −44% in PY+ and PO+, respectively); furthermore, leaves of the same order had a significantly reduced area and length compared to P− plants. PO+ plant growth was always more inhibited than PY+ growth, although no significant differences were observed.

**Figure 6:**
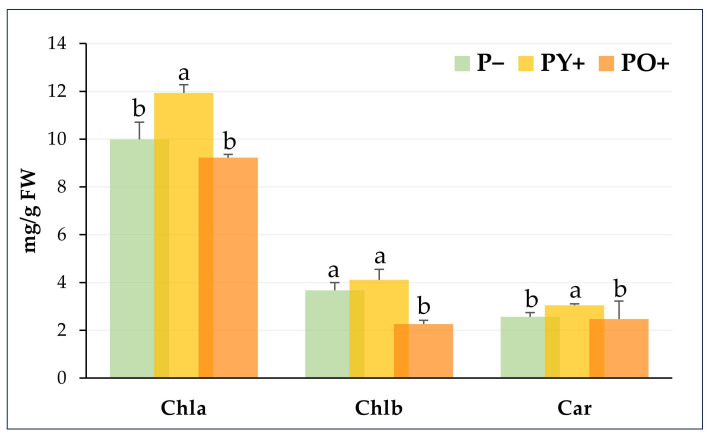
Chlorophyll (a and b) and carotenoid content of Artemisia plants grown under a transparent glass panel as control (P−), a yellow perovskite panel (PY+), and an orange perovskite panel (PO+). Bars show mean and standard deviations of 5 biological replicates with the use of the youngest fully developed leaves for each plant. Bars with different letters are significantly different at *p* ≤ 0.05 (Tukey test, *p* ≤ 0.05).

Figure 6 shows the Chla, Chlb and Car contents of Artemisia plants grown underneath PY+ and PO+ panels compared to a transparent glass panel as control (P−).

In Artemisia, a significant increase in Chla and Car was observed in plants grown under PY+ panels; PO+ plants showed values of photosynthetic pigments comparable to or, in the case of Chlb, lower than control plants. The Chla/Chlb ratio was similar to control in PY+ plants (about 2.8), while it increased up to 4.08 in PO+ plants. These results indicate a different response between tomato and Artemisia, confirming the specific adaptation of different plants to the reduced light conditions [17]. In this case, such a different response could be due to the greater resilience of the officinal plant Artemisia, more used to possible natural environmental variations than crop plants. Overall, Artemisia plant growth was reduced by the presence of both yellow and orange perovskite panels, confirming similar effects in comparison with tomato plants; nevertheless, for both plant species, the application of orange perovskite panels seemed more invasive.

### 2.4. Principal Component Analysis (PCA)

**Figure 7:**
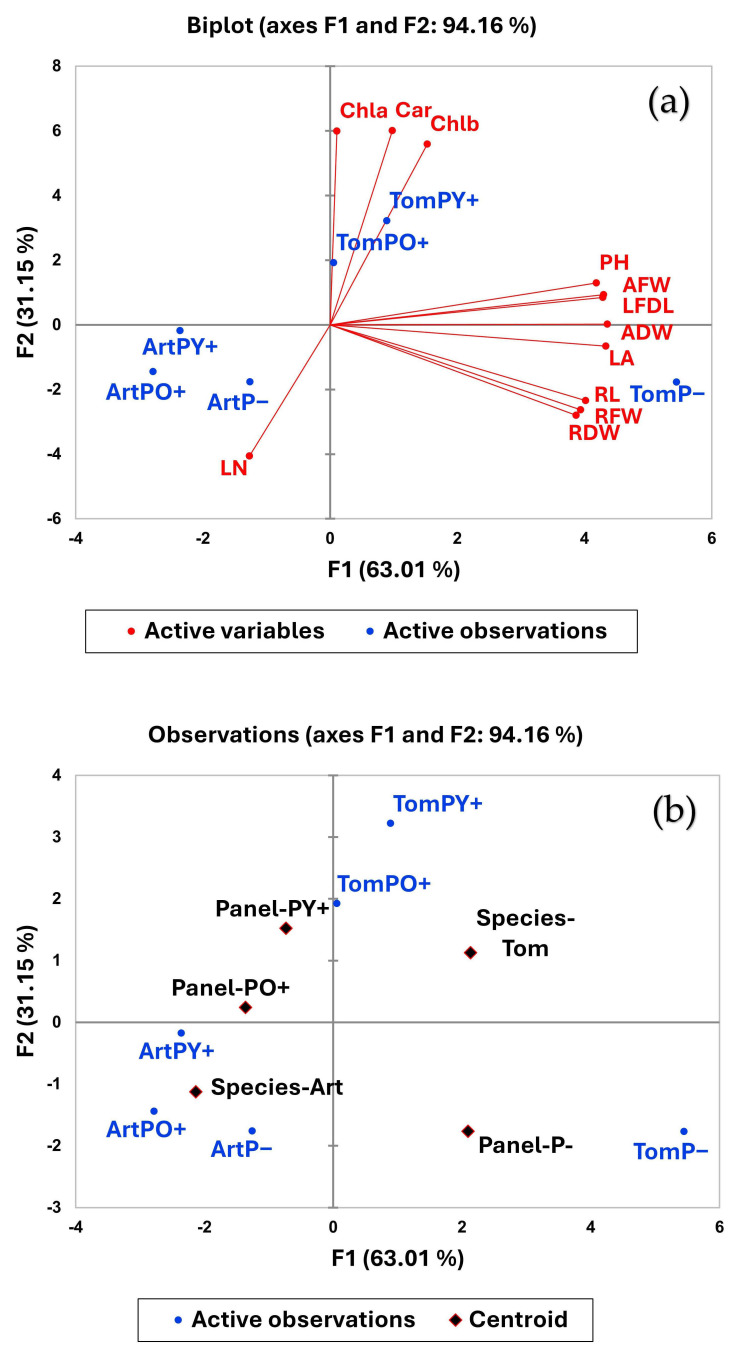
Principal Component Analysis (PCA). (**a**): Plot of PCA describing the relationship between active variables (growth parameters and pigment content) in Artemisia (Art) and tomato (Tom) plants, grown under a transparent glass panel as control (P−), a yellow perovskite panel (PY+) and an orange perovskite panel (PO+). PH: plant height; AFW: aboveground fresh weight; ADW: aboveground dry weight; LN: leaf number; LA: leaf area; LFDL: leaf fully developed length; RL: root length; RFW: root fresh weight; RDW: root dry weight. (**b**): Plot of principal component analysis with centroids (red rhombus).

To better evaluate the statistical significance of the obtained results in the two species, tomato and Artemisia, by applying two different perovskite panels, PCA analysis was performed. The results obtained for both species—tomato and Artemisia—are presented as score plot and loading plot in Figure 7a,b.

Figure 7a shows that the maximum variance described by PC1 and PC2 is 94.16% of the total variance. The first principal component (PC1) alone explains about 63.01% of the gross variability among samples and was mainly due to both root [length (RL), fresh weight (FW) and dry weight (DW)] and the aboveground plant organs’ [plant height (PH), above-ground fresh weight (AFW) and dry weight (ADW), leaf area (LA) and leaf length (LFDL)] growth-related traits. The growth parameters were positively correlated with each other and contributed to the divergence of species. Artemisia and tomato presented very different growth characteristics, which were also evident in the control plants, arranged in opposite positions on the graph.

However, the use of the panel also affected plant growth. Considering the two species separately, it was observed that in the test on tomato plants, P−, and to a lesser extent PY+, were placed near the high PC1 values, exhibiting higher growth and biomass compared to PO+. In experiments with Artemisia plants, ArtO+ grouped on the opposite end of PC1, showing much lower trait values, indicating more limited growth.

The second principal component, PC2, accounted for 31.15% of the overall variability and highlighted differences in pigment-related traits, particularly chlorophyll A (CLA), chlorophyll B (CLB), and carotenoids (CAR). These variables play a crucial role in distinguishing between species and growth conditions based on the type of panel used, pointing to variations in photosynthetic efficiency.

Figure 7b represents the principal component analysis (PCA) with centroids, which emphasizes differences due to the plant species and panels used, by summarizing their characteristics into centroid points, representing the average profile for each group.

The PCA with centroids successfully captures clear separations between the tomato and Artemisia species. Tomato species is associated with higher growth-related trait values, while Artemisia species clusters around lower values, emphasizing their distinct physiological profiles. Discrimination between control plants and plants grown under perovskite panels is also evident and in particular, it highlights an effective separation of plants grown under the yellow panel (PY+) from plants grown under the orange panel (PO+).

## 3. Materials and Methods

### 3.1. Perovskite Panels, Optical Apparatus and Light Source

Lead-free Cs2AgBiBr6 perovskite films have been synthesized by means of an innovative hydro liquid process, free of chemical solvents, mixing the precursor salts CsBr, AgBr and BiBr3 in stoichiometric and sub-stoichiometric composition for the orange and the yellow perovskite, respectively [26]. The perovskites were, successively, dropped and uniformly dispersed, by means of a brush, on supports/panels, mainly consisting of glasses, and/or indium tin oxide (ITO)/glass. The perovskite films were dried in air. The drying process was accelerated by exposure of the covered panels to a heat source of approximately 40 °C. The Cs2AgBiBr6 perovskite films were synthesized and assembled on ITO/glass at CNR-ISM, Roma, Italy.

The panels, thus, were uniformly covered with a thin film of yellow and orange Cs_2_AgBiBr_6_ perovskites, and delivered to CNR-ISPA, Lecce, Italy, for greenhouse assembly. Different support sizes, as small as (2.5 × 2.5) cm^2^ and as large as (14 × 14) cm^2^, were prepared to be placed on the roofs of greenhouses, for in vivo experiments, in tomato and Artemisia plant cultivation.

The optical properties, including transmittance, reflectance, and absorbance measurements, from both yellow and orange Cs_2_AgBiBr_6_ perovskites, were obtained using a UV-VIS (SHIMADZU UV-2700/MPC-2700, Shimadzu Europe GmbH, Duisburg, Germany) apparatus.

The light source used in indoor cultivation conditions consisted of a panel with six 36 W fluorescent lamps (Radium Spectralux^®^Plus, Radium Lampenwerk GmbH, Wipperfürth, Germany) with alternating very warm (2700 K, 3350 lm), warm (3000 K, 3350 lm), and cool white (6500 K, 3250 lm) at about 80 cm from the plant support floor.

### 3.2. Preparation of Plant Material

Seeds of *Solanum lycopersicum* L., cultivar Micro Tom [43] and *Artemisia annua* L., cultivar Artemis (Mediplant, Conthey, Switzerland) open pollinated, were surface sterilized with 70% ethanol (Sigma Aldrich, Milan, Italy) for 5 min and then thoroughly washed with sterile distilled water. After incubation in 5% sodium hypochlorite for 2 h, the seeds were rinsed 3 times in sterile distilled water to remove all traces of the sterilant and then sown in pots containing moist soil and vermiculite in the ratio of 2:1, which was moistened with a growth solution. Two weeks after germination, plantlets were transferred into the mini-greenhouses, according to the following experimental design.

### 3.3. Experimental Design

The activities were carried out indoors in a growth chamber with controlled conditions: temperature 26 °C, 16 h light/8 h dark photoperiod, by fluorescent lamps (Radium Spectralux 36W/840, Radium Lampenwerk GmbH, Wipperfürth, Germany), at several wavelengths to reproduce white light of different color temperature, and 35% relative humidity.

**Figure 8:**
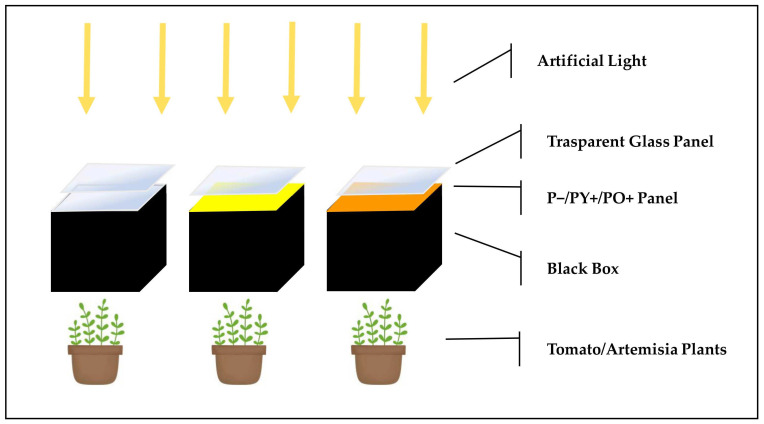
Schematic representation of the indoor experimental conditions used to evaluate the stress effect of the perovskite panels on the growth of tomato/artemisia plants. P− represents the transparent glass panel used as a control; PY+ represents the yellow perovskite panel; PO+ represents the orange perovskite panel. The artificial irradiation light from fluorescence lamps (yellow arrows) arrived from the top side at normal incidence.

Figure 8 reports the schematic representation of the experimental indoor setting. Plant pots were placed in mini-greenhouses, which consisted of dark-walled boxes covered with a yellow perovskite panel (PY+), an orange perovskite panel (PO+) or a glass panel as control (P−). Each box was covered by a transparent glass (see Figure 8). Plants were manually irrigated by pouring 5 mL of water every 3 days.

Each experiment had a total of 12 tomato/Artemisia plants with 4 plants per treatment. The experiments were repeated 5 times for both tomato and Artemisia. After 21 days, plants were collected and analyzed for growth parameters and photosynthetic pigment contents.

### 3.4. Plant Growth Parameters

Plants were collected at the end of the experiment, separating the aerial part and the roots.

Plant height was measured using a vernier caliper (±0.2 mm). Leaf number was counted visually, excluding cotyledonary leaves. Image J 1.53v software was used to measure leaf length and area, and root length. Plant aerial parts were cut into smaller pieces to make homogeneous samples for further analyses.

The aerial tissues and root aliquots were separately dried in a 65 °C oven until a constant weight (≈48 h). Fresh weight (FW) and dry weight (DW) of biomass were determined by means of analytical balance (Ohaus Pioneer Precision Balance).

### 3.5. Photosynthetic Pigment Content

Photosynthetic pigment quantification was carried out in lyophilized aerial plant tissues, according to the methodology reported in Ref. [44], with a slight modification. Briefly, samples (10 mg) were placed in a 15 mL tube, and photosynthetic pigments were extracted by adding 5 mL of DMSO. All samples were sonicated for 5 min and then placed in a water bath at 65 °C for 90 min. After centrifugation (Beckman Coulter Allegra X-15R, Beckman Coulter, Brea, CA, USA) for 10 min at 4500× *g* at 15 °C, the supernatants were transferred to a second tube and the pellet was re-extracted with DMSO. The supernatants of both extractions were pooled and centrifuged for 10 min at 3000× *g* and 15 °C, before photometric determination, by means of a microplate reader (Infinite M200 Tecan, Mannedorf, Switzerland) at 648 nm, 665 nm, and 700 nm. Absorbance was converted into a 1 cm^−1^, pathlength corrected absorbance according to Ref. [45].

The following equations were used to determine the concentrations (μg/mL) of Chl a, Chl b, and Car:

Chl a = 12.47 A_665_ − 3.62 A_649_

Chl b = 25.06 A_649_ − 6.5 A_665_

Car = (1000 A_470_ − 1.29 Chl a − 53.78 Chl b)/220

Photosynthetic pigment concentration was expressed as μg/g DW.

### 3.6. Statistical Analysis

Statistical analysis was based on a one-way ANOVA test, and Tukey’s post hoc method was applied to establish significant differences (*p* ≤ 0.05) between plants grown under different panels. Statistical analysis was performed by Sigma Plot software version 11.0 (Systat Software, Inc. San Jose, CA, USA). Each data point was the mean of ≥3 replicates ± standard deviation (SD). Principal component analysis (PCA) was used to analyze the relationship between growth parameters of tomato and Artemisia plants under three light conditions. Factor reduction analysis was performed on the indicated parameters by means of a correlation matrix for the determination of the principal components using the statistical software XL Stat Software, version 2022.1 (Addinsoft, Paris, France).

## 4. Conclusions

In summary, the results highlight, for the first time, that the use of both yellow and orange perovskite panels, though filtering incident radiation, did not prevent plant growth, allowing the photosynthesis-specific radiation to reach the plants cultivated underneath. However, differences were observed between yellow and orange panels, orange being more impactful to some growth parameters.

Both plant species, tomato and Artemisia, could adapt to the reduced light availability due to perovskite panels by increasing the content of photosynthetic pigments. Nevertheless, differences, also confirmed by statistical analysis, were found between tomato and Artemisia, confirming the species-specific adaptation to the reduced light conditions. This suggests the need to identify the most proper plant species, also in view of using photovoltaic panels based on semi-transparent perovskites in outdoor conditions under sunlight.

The next step of this study, including outdoor cultivation, is in progress and will help assess the suitability of these semi-transparent perovskite panels to ensure crop yield and quality.

## Figures and Tables

**Table 1:** Growth parameters in tomato plants under a transparent glass panel as control (P−), a yellow perovskite panel (PY+) and an orange perovskite panel (PO+) after 21 days. The data indicate means ± SD from five independent experiments. Means within a column with different letters are significantly different (Tukey test, *p* ≤ 0.05).

Panel	AERIAL PARTS		
	Plant Height (cm)	Fresh Weight (g/Plant)	Dry Weight (g/Plant)
P−	13.38 ± 1.65 a	4.38 ± 0.32 a	0.448 ± 0.033 a
PY+	10.25 ± 0.87 b	2.46 ± 0.19 b	0.201 ± 0.016 b
PO+	8.15 ± 0.56 c	1.89 ± 0.31 c	0.162 ± 0.017 c
	**ROOTS**		
	**Length** **(cm)**	**Fresh Weight** **(g/plant)**	**Dry Weight** **(g/plant)**
P−	9.48 ± 0.86 a	0.646 ± 0.053 a	0.0806 ± 0.0070 a
PY+	3.83 ± 0.29 b	0.067 ± 0.005 b	0.0054 ± 0.0004 b
PO+	3.39 ± 0.39 b	0.047 ± 0.003 b	0.0037 ± 0.0003 b

**Table 2:** Leaf parameters of tomato plants grown under a transparent glass panel as control (P−), a yellow perovskite panel (PY+), and an orange perovskite panel (PO+) after 21 days. The data indicate means ± SD from five independent experiments. Means within a column with different letters are significantly different (Tukey test, *p* ≤ 0.05).

Panel	Leaf Number	Leaf Area (cm^2^)	Length of Fully Developed Leaf (cm)
P−	5.67 ± 0.58 a	12.86 ± 1.38 a	9.15 ± 0.66 a
PY+	4.51 ± 0.52 b	5.54 ± 0.74 b	6.61 ± 0.39 b
PO+	3.83 ± 0.51 c	4.52 ± 0.36 b	6.21 ± 0.35 b

**Table 3:** Growth parameters, plant height, fresh and dry weight for aerial part; length, fresh and dry weight for plants root, in Artemisia plants under a transparent glass panel as control (P−), a yellow perovskite panel (PY+), and an orange perovskite panel (PO+) after 21 days. The data indicate means ± SD from five independent experiments. Means within a column with different letters are significantly different (Tukey test, *p* ≤ 0.05).

Panel	AERIAL PARTS		
	Plant Height (cm)	Fresh Weight (g/Plant)	Dry Weight (g/Plant)
P−	6.46 ± 0.45 a	0.559 ± 0.045 a	0.082 ± 0.008 a
PY+	3.66 ± 0.16 b	0.123 ± 0.013 b	0.013 ± 0.008 b
PO+	2.91 ± 0.18 c	0.101 ± 0.009 b	0.009 ± 0.001 b
	**ROOTS**		
	**Length** **(cm)**	**Fresh Weight** **(g/plant)**	**Dry Weight** **(g/plant)**
P−	4.25 ± 0.33 a	0.136 ± 0.084 a	0.0158 ± 0.0082 a
PY+	3.11 ± 0.29 b	0.022 ± 0.002 b	0.0031 ± 0.0002 b
PO+	2.83 ± 0.32 b	0.012 ± 0.001 b	0.0012 ± 0.0009 b

**Table 4:** Leaf parameters in Artemisia plants grown under a transparent glass panel as control (P−), a yellow perovskite panel (PY+) and an orange perovskite panel (PO+) after 21 days. The data indicate mean ± SD from five independent experiments. Means within a column with different letters are significantly different (Tukey test, *p* ≤ 0.05).

Panel	Leaf Number	Leaf Area (cm^2^)	Length of Fully Developed Leaf (cm)
P−	10.25 ± 0.75 a	3.48 ± 0.47 a	4.79 ± 0.52 a
PY+	6.82 ± 0.53 b	1.62 ± 0.18 b	3.58 ± 0.27 b
PO+	5.78 ± 0.93 b	1.49 ± 0.29 b	3.38 ± 0.24 b

## Data Availability

All data that support the findings of this work are included within the article.
